# Current carried by the Slc26 family member prestin does not flow through the transporter pathway

**DOI:** 10.1038/srep46619

**Published:** 2017-04-19

**Authors:** Jun-Ping Bai, Iman Moeini-Naghani, Sheng Zhong, Fang-Yong Li, Shumin Bian, Fred J. Sigworth, Joseph Santos-Sacchi, Dhasakumar Navaratnam

**Affiliations:** 1 Dept. of Neurology, Yale School of Medicine, 333 Cedar Street, New Haven, CT, 06510 USA; 2 Dept. of Surgery, Yale School of Medicine, 333 Cedar Street, New Haven, CT, 06510 USA; 3 Yale Center for Analytical Sciences, Yale School of Public Health, 300 George St., Ste Suite 555, New Haven, CT 06511, USA; 4 Dept. of Cellular and Molecular Physiology, Yale School of Medicine, 333 Cedar Street, New Haven, CT, 06510, USA; 5 Dept. of, Neuroscience, Yale School of Medicine, 333 Cedar Street, New Haven, CT, 06510, USA

## Abstract

Prestin in the lateral membrane of outer hair cells, is responsible for electromotility (EM) and a corresponding nonlinear capacitance (NLC). Prestin’s voltage sensitivity is influenced by intracellular chloride. A regulator of intracellular chloride is a stretch-sensitive, non-selective conductance within the lateral membrane, G_metL_. We determine that prestin itself possesses a stretch-sensitive, non-selective conductance that is largest in the presence of thiocyanate ions. This conductance is independent of the anion transporter mechanism. Prestin has been modeled, based on structural data from related anion transporters (SLC26Dg and UraA), to have a 7 + 7 inverted repeat structure with anion transport initiated by chloride binding at the intracellular cleft. Mutation of residues that bind intracellular chloride, and salicylate treatment which prevents chloride binding, have no effect on thiocyanate conductance. In contrast, other mutations reduce the conductance while preserving NLC. When superimposed on prestin’s structure, the location of these mutations indicates that the ion permeation pathway lies between the core and gate ring of helices, distinct from the transporter pathway. The uncoupled current is reminiscent of an omega current in voltage-gated ion channels. We suggest that prestin itself is the main regulator of intracellular chloride concentration via a route distinct from its transporter pathway.

Outer hair cell (OHC) electromotility is at the heart of cochlear amplification, a mechanism responsible for the mammal’s ability to hear sounds of extremely low intensity^1^,^2^. Prestin is a member of the SLC26 anion transporter family that now includes 10 members, and was initially identified in a search for the OHC lateral membrane motor^3^,^4^,^5^. Subsequent experiments have confirmed that prestin (SLC26a5) is required for electromotility and has all the properties including electromotility (EM) and non-linear capacitance (NLC) of the voltage-driven motor in OHCs^6^,^7^,^8^,^9^, expanding and contracting up to several thousand Hz^10^. Recent modeling and experimental data have established that it most likely has a structure similar to the distantly related bacterial uracil transporter UraA^11^,^12^, and this is now corroborated by the crystal structure of the more closely related bacterial homologue SLC26Dg^13^. This modeling experiment, that shows prestin with a 7 + 7 inverted repeat structure, supersedes previous models suggesting similarity of the SLC26 family to the ClC bacterial chloride transporter/ion channel family^12^,^14^,^15^.

Intracellular chloride anions play a critical role in prestin’s function^16^, setting, in an allosteric-like manner, its operating voltage range and affecting transition rates between expanded and contracted states^17^,^18^. How intracellular chloride levels are regulated in OHCs remains largely unknown. A dominant stretch-sensitive, non-selective conductance, G_metL_, has been observed in the lateral membrane of OHCs, and awaits molecular identification^19^. An obvious candidate would be prestin itself, although its ability to transport anions has been controversial. An initial model suggested a complete absence of transmembrane ion flux; instead, chloride movement in a truncated cycle of transport was hypothesized to act as the protein’s voltage sensor^16^. Many subsequent observations have challenged this hypothesis^17^,^19^,^20^,^21^,^22^,^23^,^24^. Additionally, experiments have shown formate and oxalate uptake based on isotopic flux^20^ (although one report failed to do so^25^), and HCO_3_^−^/Cl^−^ exchange at a 2:1 ratio generating small transporter currents^26^. Interestingly, large currents associated with prestin expression arise in the presence of the pseudohalide thiocyanate, similar to uncoupled currents in SLC26a6 and SLC26a3^14^,^15^,^27^. In this paper we show that prestin itself has a tension-sensitive, non-selective leakage conductance similar to G_metL_ that operates independently of its putative transporter pathway.

## Results

**Figure 1:**
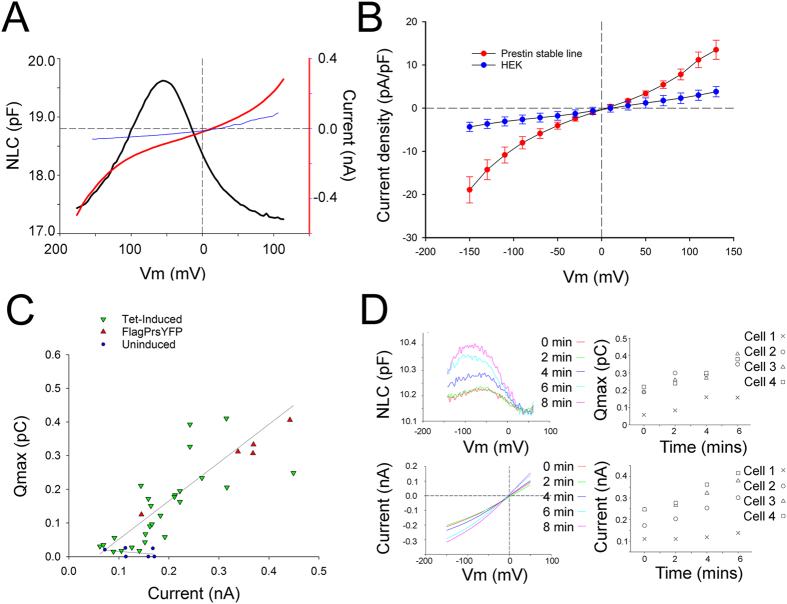
Prestin expression induces a current. (**A**) Voltage dependence of gating charge (NLC) and current, recorded in the presence of Cl^−^ after tetracycline induction of prestin expression in a HEK cell. There is a large non-linear capacitance (left axis, with a Q_max_ of 302 fC, z of 0.81e) accompanied by a voltage dependent current (right axis, current, in red, of 0.64 nA for voltage range of −160 mV to 110 mV. In blue is the current of a control cell). (**B**) I-V relationship of average currents of cells after tetracycline induced prestin expression (n = 16) is compared to native HEK cells (n = 10). Prestin expression is associated with a significant increase in voltage dependent current (p < 0.001, mixed model analysis for correlated measures). Pipette solution contained 100 mM KCl, 20 mM TEACl, 20 mM CsCl, 2 mM CoCl_2_, 2 mM MgCl_2_, 5 mM Hepes, pH 7.2 and the bath solution contained 100 mM NaCl, 20 mM CsCl, 5 mM EGTA, 2 mM MgCl_2_, 10 mM Hepes, pH 7.2. Error bars are standard error. (**C**) There is a linear relationship between size of prestin induced currents and NLC. The difference in current at 110 mV and −150 mV is plotted against total charge movement (*Q*_max_) of individual cells. Green triangles are cells after tetracycline induction (n = 27), red triangles are from cells stably expressing high levels of prestin (n = 5) and circles are currents from untransfected HEK cells (n = 6). The line represents a simple linear regression in tetracycline induced cells (R = 0.78, n = 27). D. Increased surface expression of prestin after Golgi release is temporally associated with an increase in current. Inducible cell lines were maintained at 22 °C for 24 hours to allow accumulation of prestin in the Golgi immediately after tetracycline induction. Cells were then continuously recorded using a two-sine protocol and the bath temperature raised to 37 °C. There is increasing prestin expression on the surface of the cell evidenced by time dependent increase in NLC (upper left, single cell; upper right, four cells) that is correlated with increasing currents (lower left, single cell; lower right, four cells).

Schänzler and Fahlke^27^ demonstrated an uncoupled conductance for SCN^−^ ions that correlates with the expression of prestin. We find that expression of prestin induces currents in the absence of SCN^−^, as well. In the presence of Cl^−^, we observed a current in tet-inducible, prestin expressing HEK cells^28^, which is significantly larger than currents in untransfected or uninduced HEK cells (Fig. 1A,B). The current, several hundred pA per cell in symmetrical chloride solutions, shows a non-linear voltage dependence, similar to the non-selective current (I_metL_) observed within the lateral membrane of OHCs^29^. The size of the current correlates with the magnitude of prestin-associated, voltage-dependent NLC, the electrical signature of OHC mechanical activity, indicating that the current is associated with prestin successfully expressed on the surface of the HEK cell (Fig. 1C). Further evidence that prestin carries this current is shown by the parallel increases of current and NLC magnitudes upon delivery of prestin to the plasma membrane after release of low-temperature Golgi block (Fig. 1D)^30^. This effect was clearly demonstrated in 8 cells (four cells are shown in Fig. 1D).

**Figure 2:**
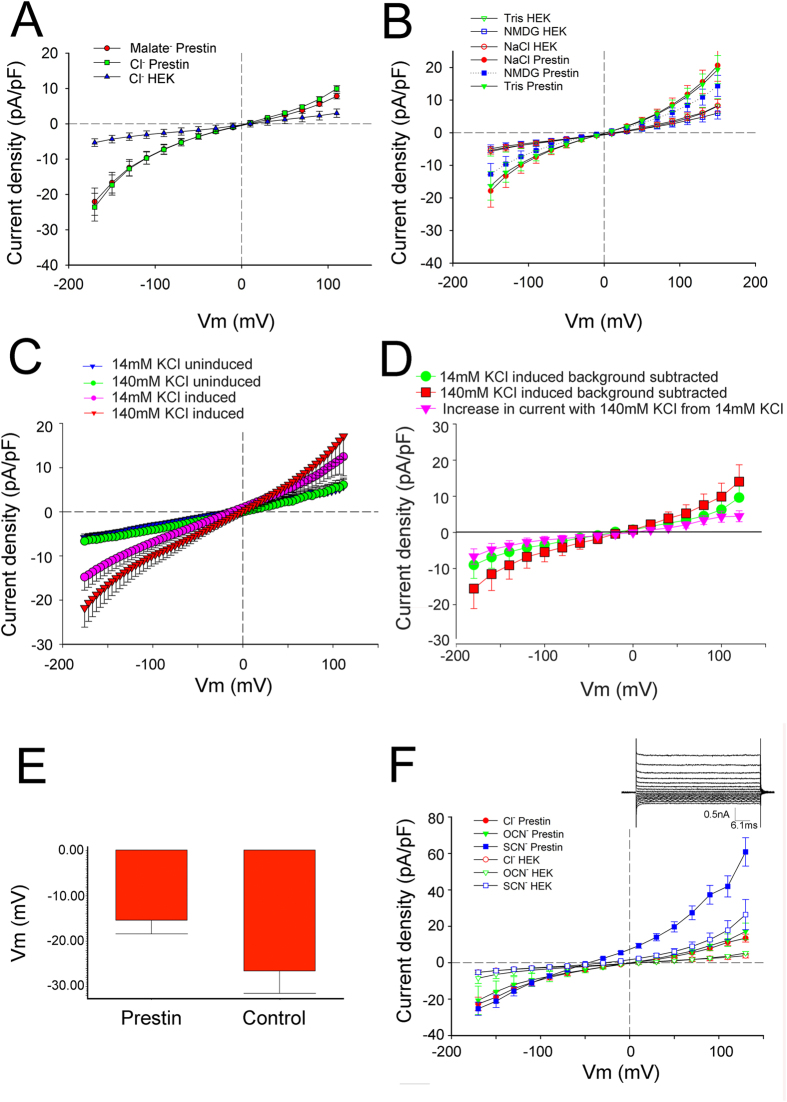
Prestin expression is associated with a non-selective current that is augmented with thiocyanate. (**A**) Substitution of extracellular NaCl with NaMalate did not affect the size of the current (P > 0.05, n = 7) or affect the reversal potential. (**B**) Extracellular substitution of Na^+^ with NMDG^+^ significantly reduced the size of the currents (P < 0.001, n = 10). In contrast, substitution with Tris^+^ did not affect the current size or shift reversal potential. (**C**) Prestin associated currents are non-selective. In prestin expressing cells the substitution of 14 mM extracellular KCL with 140 mM KCL, while maintaining pipette (intracellular) KCL at 140 mM, results in an increase in the size of the whole-cell current at both depolarizing and hyperpolarizing voltages (p < 0.05, n = 7; +/−SEM). In induced cells the reversal potential is 1.2 mV (+/−3.6, n = 7) with symmetrical 140 mM KCl and −14.7 mV (+/−3.9, n = 7) with 14 mM extracellular KCl. These data are consistent with permeability ratios for K^+^: Cl^−^ of 1: 0.45. (**D**) Subtraction of baseline currents in uninduced cells from currents after prestin induction confirms the non-selective nature of the prestin associated current. (**E**) Resting membrane potentials recorded in current clamp mode immediately after achieving the whole cell configuration show significant depolarization in prestin expressing cells to −15.4 mV (+/−2.9 SE, n = 11) compared to −26 mV (+/−4.8 SE n = 7) in uninduced HEK cells (p < 0.05, students t test). (**F**) I-V relationships show a non-linear voltage dependent current with cyanate and thiocyanate. Average currents corrected for the size of the cell after induced expression of prestin in HEK cells are compared with currents in uninduced HEK cells before and after perfusion with 100 mM NaOCN, and 100 mM NaSCN (+/−SE). Currents increase significantly in prestin expressing cells compared to HEK cells in the presence of extracellular Cl^−^ (P < 0.001, n = 16 prestin, n = 5 HEK), OCN^−^ (p < 0.001, n = 3 prestin, n = 5 HEK) and SCN^−^ (p < 0.001, n = 17 prestin, n = 5 HEK) with the largest effects seen with SCN^−^. Inset: Thiocyanate current activates instantaneously and shows no deactivation. Prestin currents were recorded in a cell perfused with 100 mM NaSCN (holding at 0 mV, −150 to +150 mV with 20 mV steps of 50 ms duration).

Absence of ion selectivity of the conductance was shown by substitution of anions and cations in both the intracellular and extracellular solutions; no substitutions resulted in clear change in the size of these currents, except for the large cation NMDG^+^ (Fig. 2A, B). Moreover, there was no change in the I-V relationships (Fig. 2A, B). The anion substitution data also suggest that the current was uncoupled: extracellular substitution of Cl^−^ with the anion malate did not affect the size of the current or its reversal potential (Fig. 2A). A similar effect was seen with oxalate, IO_3_, and methanesulfonate (data not shown). Each of these ion replacements is known to cause a shift of up to 50 mV in the NLC voltage operating range^17^. Similarly, substitutions of intracellular and extracellular K^+^ and Na^+^ with other ions (Na^+^, K^+^ or Tris^+^) did not affect the size of the current or its reversal potential (Fig. 2B). In contrast, when extracellular Na^+^ was replaced with NMDG^+^, we note a significant decrease in the size of the current (Fig. 2B). NMDG^+^ substitution affected both outward and inward currents, as might be expected from a pore-block mechanism. Further confirming the non-selective nature of the conductance, we observed a decrease in the size of the current at both depolarizing and hyperpolarizing voltages when extracellular KCl was decreased from 140 to 14 mM (the pipette solution contained 140 mM KCl, setting the intracellular concentrations) while 300 mOsm extracellular osmolarity was maintained with sucrose. The reversal potential shifted by −15 mV +/− 2 mV with 14 mM extracellular KCl, and is consistent with a calculated permeability ratio of 0.45 for Cl^−^ relative to K^+^ (2C,D). Consistent with the current being carried by a non-selective “open pore”, we note that cells expressing prestin show significant depolarization, relative to controls, in resting membrane potential, determined immediately after establishing the whole cell configuration under current clamp (Fig. 2E).

Similar to the Schänzler and Fahlke observation^27^, we find larger uncoupled currents (nA magnitude) in prestin-expressing cells in the presence of thiocyanate (Fig. 2F), also akin to the effect observed in Slc26a3 and Slc26a6^14^,^15^,^27^. As expected for a current predominantly carried by SCN^−^, the current was directed outward with extracellular thiocyanate and inward with intracellular thiocyanate. The reversal potential with extracellular SCN^−^ (in millimolar: 100 SCN^−^ and 44 Cl^−^ in the bath; 122 Cl^−^ in the pipette with Na^+^ as the counterion in the pipette and bath solutions), was −29 ± 2 mV. We calculate a permeability ratio of 0.27 for Na^+^ and 0.11 for Cl^−^ relative to SCN^−^. These permeability ratios are underestimates, and most probably they are discordant due to the background conductances that are also seen in control HEK cells (Fig. 2).

**Figure 3:**
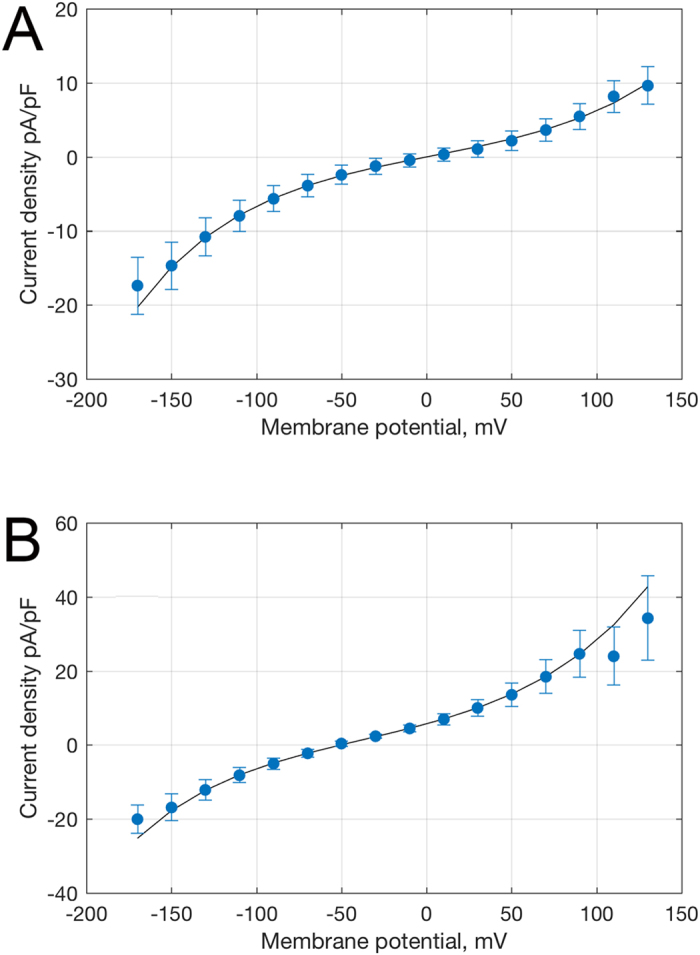
Single-barrier model I-V fits. Plotted is whole-cell current density in prestin-induced cells (n = 19) after subtraction of the corresponding current in control HEK cells (n = 19) in (**A**) symmetrical 140 mM KCl^−^ or (**B**) with extracellular SCN^−^ replacing Cl^−^ (+/−SEM). Curves are fitted with a single-barrier current-voltage relationship.

In the presence of thiocyanate, voltage step-induced currents were instantaneous in onset and showed no deactivation (Fig. 2F, inset). We observe a similar instantaneous onset with no deactivation with Cl^−^ substitution (data not shown). The shape of the I-V relationship is similar to that observed with Cl^−^ as the dominant intra and extracellular anion, with increasing slope upon both depolarization and hyperpolarization. This I-V relationship would be expected from a channel having a large central energy barrier and whose currents are sufficiently small to exclude diffusion limitation. A symmetrical, single-barrier channel model, which yields a hyperbolic sine I-V relationship, fits the data well (Fig. 3) but with a reduced voltage dependence, obtained by setting the valence to 0.75 instead of unity. This effect could arise from a broadened central barrier, or from the presence of barriers at slightly different locations for different ion species and polarities. According to the fitted model with external SCN^−^ and internal Cl^−^ the ratio of permeabilities *P* ≈ 6.

**Figure 4:**
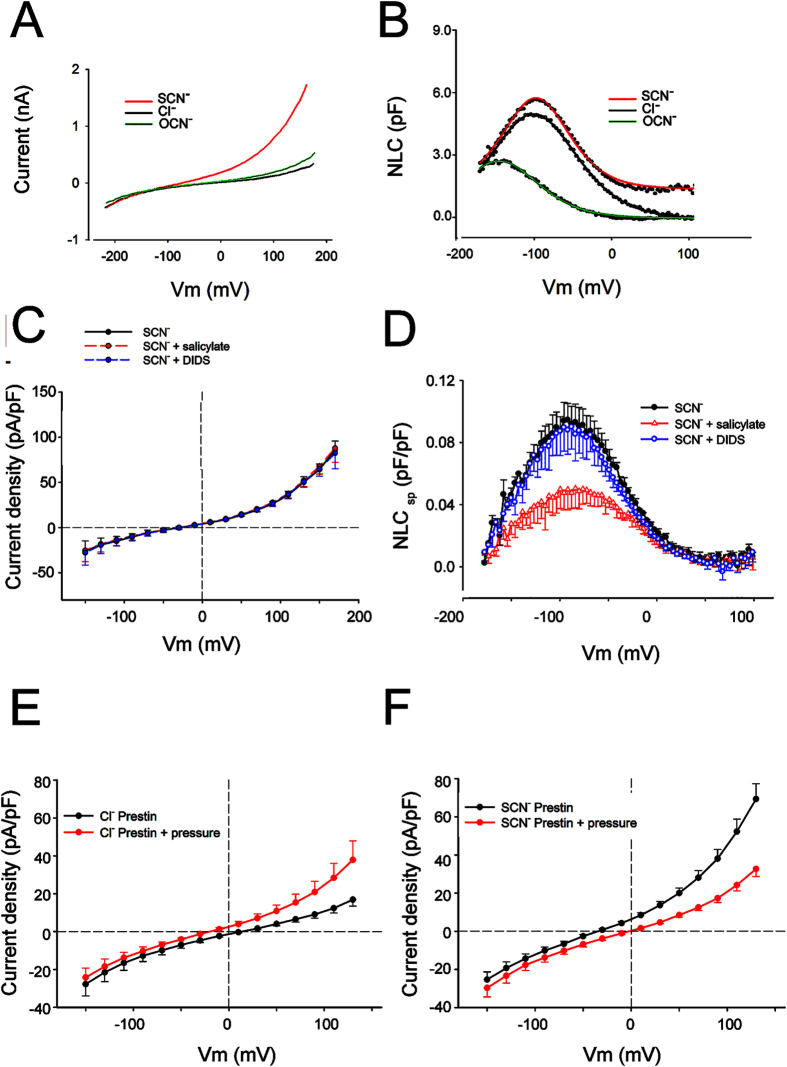
Currents and NLC associated with prestin are separable. (**A,B**). Shown are NLC (**A**) and currents (**B**) in a single cell perfused with extracellular Cl^−^, OCN^−^ and SCN^−^. SCN^−^ increases current significantly and affects gating charge, while OCN^−^ causes a slight increase in current while causing a hyperpolarizing shift in *V*_h_. (**C**,**D**) Extracellular salicylate (20 mM) has no effect on currents (**C**) in the presence of extracellular SCN^−^, while reducing NLC (**D**). 1 mM DIDS does not affect NLC or SCN^−^ current. Shown are average tracings from three cells (+/−SEM). (**E**,**F**) The effects of fluid-jet pressure on the size of the current are dependent on the conducting anion. With Cl^−^ in the bath solution there is an increase in the size of the current with exposure of the cell to a fluid jet (**E**). In contrast, in the presence of extracellular SCN^−^, the size of the current decreases with exposure to a fluid jet (**F**). Shown are average tracings from 10 cells (+/−SEM).

As shown in Figs 2 and 4, a similar but smaller whole-cell current was observed when cyanate replaced thiocyanate. These anion substitutions were notable for effects on the Boltzmann parameter *V*_h_ of NLC: while small shifts in *V*_h_ were noted when switching from chloride to thiocyanate, much larger effects on *V*_h_ were found with cyanate; on the other hand, significant effects on Qmax were found with both anions (Fig. 4B, Table 1).

Notably, currents in the presence of thiocyanate and extracellular Cl- were unaffected by application of salicylate that, however, eliminated NLC (Fig. 4C,D, and Supplementary Figure 1). These data argue against a transporter mechanism as the origin of the current since salicylate binds to the intracellular Cl- binding site and has been shown to reduce transporter currents^31^ and inhibit NLC^32^,^33^. Consistent with this observation, the use of 1 mM DIDS, the well-established blocker of anion transport^34^,^35^,^36^, did not affect the size of these currents in the presence of thiocyanate, nor did it affect NLC (Fig. 4C,D). Application of membrane tension by exposing the cell to a fluid jet of extracellular solution resulted in a decrease in the size of the current in the presence of thiocyanate and an increase in the size of the current in the presence of chloride and cyanate (Fig. 4E,F, for comparison see Supplementary Figure 2 for the size of effects of pressure on currents of HEK cells not expressing prestin). We observed a similar effect in guinea pig OHCs with large outward currents in the presence of extracellular thiocyanate that decreased in size in the presence of fluid jet stimulation (data not shown).

An estimate of the unitary current of a prestin-associated channel can be obtained as the whole-cell current normalized by the number of prestin molecules. This number can be derived from the magnitude of NLC^37^. In this way we estimate that at +50 mV the unitary currents are 0.010 fA and 0.062 fA with extracellular Cl- and thiocyanate, respectively.

Another approach to determining the size of unitary currents is fluctuation analysis. Schänzler and Fahlke^27^ observed fluctuations in thiocyanate currents having a 1/f spectral density in whole-cell recordings. Because, however, the variance of the fluctuations had no clear relationship to the mean current, they concluded that the unitary currents were too small to be estimated with any reliability. We chose instead to record currents in excised patches to obtain higher sensitivity. Individual patch experiments (with patches of 1–2 mm^2^) from prestin expressing cells demonstrate an increase in average current of +11.94 pA (+50 mV) and −12.56 pA (−50 mV), and +7.73 pA (+50 mV) and −10.36 pA (+50 mV) over HEK cells in the presence of thiocyanate and chloride, respectively.

**Figure 5:**
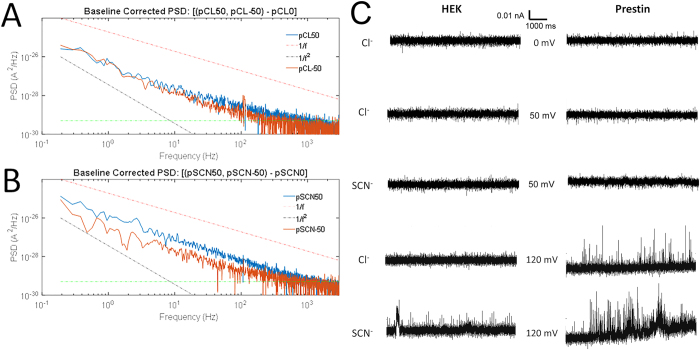
Noise analysis of currents in prestin expressing cells. (**A**) Currents in inside-out patches from prestin-expressing cells were recorded in symmetrical 140 mM Cl^−^ solutions at −50 mV (red line), 0 mV and +50 mV (blue line) for 10 seconds. Spectra from 11 patches were averaged. Spectra are plotted after subtraction of the 0 mV background spectrum. The frequency dependence is well described by a power-law decay plus a shot-noise (constant, green dotted lines) component with no apparent Lorentzian components. (**B**) Corresponding averaged, subtracted spectra obtained from same inside-out patches with 100 mM NaSCN on the intracellular side. As expected, noise is larger for currents at +50 mV where, by convention, the movement of SCN^−^ current is inward, although the size of the mean patch current at +50 mV (Table 2) is similar to that at −50 mV (Table 2). Reference lines of 1/f and 1/f^2^ slopes accompany data plots as visual aids. (**C**) Consistent with the small unitary currents estimated from the noise variance, no ion-channel events were visible except at extreme potentials such as +120 mV. Representative recordings are shown from excised inside out patches of HEK cells (left) and HEK cells with prestin expression (right) held at 0, 50 and120 mV in the presence of 140 mM Cl^−^ and 100 mM SCN^−^/40mMCl^−^.

In the presence of Cl^−^ and SCN^−^ we also observed fluctuations with a power-law frequency dependence, approximately 1/*f*, whose magnitude clearly depended on membrane potential (Fig. 5A,C). The variance of the fluctuations was computed in two steps. First, the spectral density at 0 mV applied potential was taken to be the background spectrum, and was subtracted from spectra obtained at +50 mV or −50 mV (Fig. 5B,D). The variance was then calculated as the integral of the subtracted spectrum over the frequency bandwidth of 0.1 to 1000 Hz. Very rough estimates of the unitary current can be made from the ratio of the variance to the mean current, yielding values of about ±2 fA at ±50 mV driving force in chloride solutions, and about 5 fA for outward current carried by SCN^−^ at +50 mV (Table 2).

This simple estimation of the elementary current magnitude is very approximate because it is based on several assumptions. First, it assumes switching between open and closed states, with a low probability of the open state. Second it is assumed that the fluctuations are approximately confined to the 0.1 to 1000 Hz frequency band. Finally, the use of the spectral density at 0 mV as the background estimate is not strictly correct for currents having a nonzero reversal potential. However, as the variance depends quadratically on the current, which is small at 0 mV, the error from this approximation is probably smaller than the errors arising from the other assumptions.

The power spectrum of current fluctuations in well-behaved transporters and gated ion channels typically shows one or more Lorentzian components^38^,^39^,^40^,^41^,^42^,^43^. A power-law decay, like the one observed here over a range of four orders of magnitude in frequency, is instead consistent with fluctuations having a broad distribution of relaxation times. The estimated unitary currents are far too small to be visible in patch recordings, and consistent with this, no channel events were visible in the traces recorded at ±50 mV. However, at large voltages such as +120 mV, channel-like activity could be seen (Fig. 5E). We assume that these very large currents arise from different channels than those under study here.

**Figure 6:**
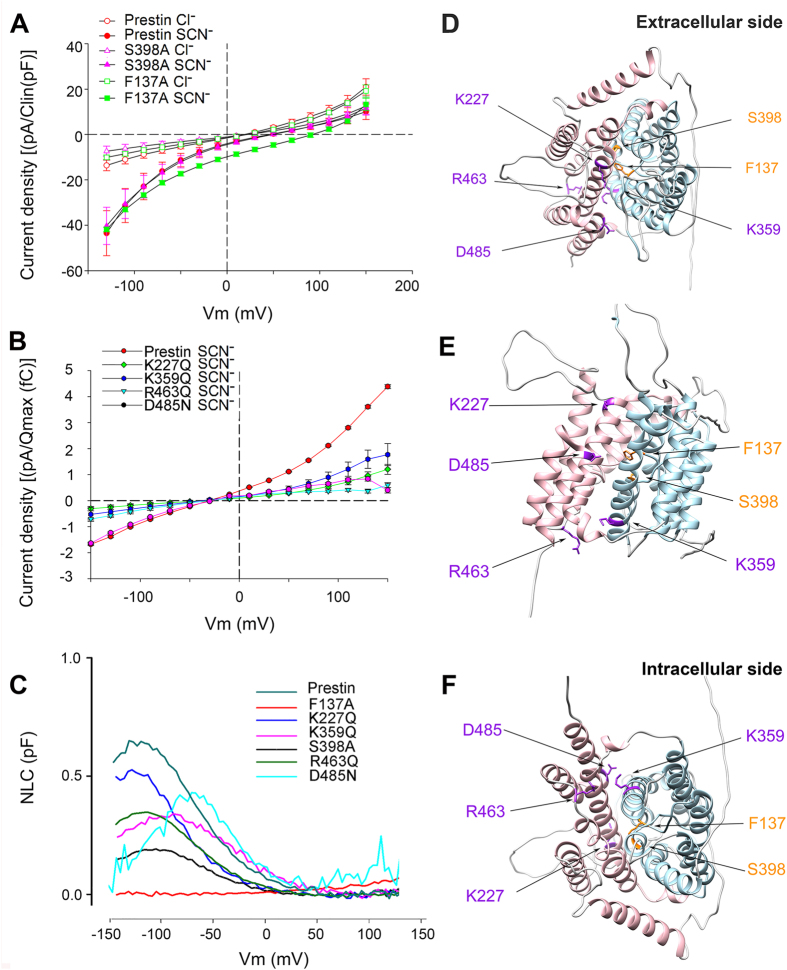
SCN^−^ currents in prestin suggest an ancillary pathway independent of the transporter pathway. (**A**) Mutation of residues critical for Cl^−^ binding, the first step in transport, has no effects on SCN^−^ currents. Average currents of CHO cells expressing prestin (n = 5), F137A (n = 4) and S398A (n = 7) show no differences (+/−SEM). Currents, after correction for background currents, were divided by linear capacitance to correct for differences in cell size and plotted against voltage (F137A did not have easily measurable NLC, so the more rigorous normalization to NLC was not possible). Cells show large inward currents in the presence of intracellular SCN^−^ (100 mM) at depolarizing voltages. Intracellular SCN^−^ was used since the residue corresponding to S398 lies closer to the inner cleft of the transporter pathway and binds to intracellular uracil in UraA; the residue corresponding to F137 acts as a barrier to the diffusion of uracil to the exterior surface. (**B**) Neutralization of charged residues lying outside the transporter pathway reduce SCN^−^ currents. Average currents of CHO cells expressing K227Q (n = 7), K359Q (n = 7), R463Q (n = 9), and D485N (n = 18) show significantly reduced currents in the presence of extracellular SCN^−^ (p < 0.001). To account for variations in expression, current averages for each mutant construct, after correction of average background currents, were normalized by the NLC charge *Q*_max_, and plotted against voltage. (**C**) NLC traces from residues (K227Q, K359Q R463Q and D485N) where mutation affected SCN^−^ currents but had measurable NLC, and of mutations in the transporter pathway (F137A and S398A). (**D**–**F**) The location of residues that line a potential accessory pathway are shown on the structure of prestin modeled on the crystal structure of the bacterial uracil transporter UraA rendered with Chimera^11^,^12^. The core domains are colored blue and the gate domains colored in pink. (**D**–**F**) are the molecule viewed from above, the side and below respectively. In the potential accessory pathway lined by alpha helices 5, 8 and 14, the contained amino acids K227, K359 and D485, and R463, that lies in the loop connecting helices 12 and 13, are colored purple. Residues modeled to bind intracellular chloride (F137 and S398) are colored orange.

To date there are scant structural data on eukaryotic Slc26 transporters. However, recently, prestin was modeled, with confirmatory molecular dynamics simulations and experimental evidence, to closely fit the crystal structure of a bacterial uracil transporter, UraA, that is distantly related to the SLC26 family^11^,^12^. A recent paper has observed an almost identical 7 + 7 inverted repeat structure in the more closely related bacterial homologue SLC26Dg, a facilitator of proton-coupled fumarate symport from Deinococcus geothermalis^13^. For our analyses, we use the 3D crystal structure of UraA as the template (see Supplementary Figure 3 for the sequence alignment using HHpred), as there are better structure-function correlates in UraA than in SLC26Dg. Several key residues in UraA that bind to uracil and are critical for transport are conserved in prestin, including S398 and F137 based on hidden Markov model homology (HHpred). Of these residues E290 in UraA, corresponding to S398 in prestin, is modeled to bind to uracil close to its intracellular cleft. Similarly, F73 in UraA, corresponding to F137 in prestin, binds to uracil and acts as a barrier for its escape to the periplasmic surface^12^. We reasoned that mutation of these residues in prestin would reduce the easily measurable thiocyanate currents if prestin were to mediate these currents via its transporter pathway. However, mutation of these residues to alanine had little effect on the size of thiocyanate currents but they did shift *V*_h_ of NLC (F137A did not have detectable NLC likely since Vh was so shifted; membrane targeting was confirmed by confocal microscopy (Supplementary Figure 4)). We interpret these data as consistent with a model of Na^+^, Cl^−^ and thiocyanate movement through the protein independent of its transporter pathway (Fig. 6A). We note that mutation of F137A did produce a positive shift in SCN^−^ reversal potential, although an increase SCN^−^ conductance is not borne out in the size of SCN^−^ currents at −130 mV. One possible explanation for the shift in reversal potential is of a decrease in cation conductance in the presence of thiocyanate.

In contrast to the absence of effects on thiocyanate or chloride currents from mutation of residues corresponding to those in the UraA binding pocket, we note marked reductions in SCN^−^ currents with charge neutralizing mutations of several charged residues in prestin’s transmembrane domains (Fig. 6B). The side chains of these residues reside at the interface between the core and gate domain (Fig. 6D–F). These mutations (K227Q, K359Q and D485N) in three widely separated alpha helical transmembrane domains 5, 8 and 14, preserved NLC (Table 3, Fig. 6C), thus confirming surface expression of functional molecules. Examining the modeled structure of prestin reveals a potential accessory pathway, which links these widely separated residues that lie in physical proximity in its tertiary structure, independent of the suspected substrate binding site between the alpha-helical regions of TM3 and 10. Indeed, this pathway allows access to the inner membrane surface while access to the external surface is partially obstructed by a loop linking transmembrane domains 3 and 4. Significantly, an additional mutation, R463Q, that also reduced SCN^−^ currents, lies in an unstructured loop linking transmembrane domains 12 and 13. It lies at the intracellular cleft of the transporter and is in a potential pathway linking the extracellular medium and the envisaged accessory pathway. Note however, that our model is based on the structure of UraA and SLC26dg both of which capture a single state, namely an inside open conformation. Determining the exact pathway of the non-selective conductance will require more structural data particularly of the outside open and other intermediary conformations.

## Discussion

Prestin has evolved to facilitate hearing in mammals by enhancing auditory thresholds by up to 1000 fold, through cochlear amplification^1^,^2^. Since it is a member of the SLC26 anion transporter family, it is no surprise that anions, chiefly intracellular chloride, control many of its biophysical characteristics^16^,^17^,^18^. It is paramount that intracellular chloride homeostasis be understood, since chloride manipulations have been shown to reversibly abolish cochlear amplification^44^. Here we present evidence that prestin, itself, provides a stretch-sensitive leakage pathway for anions that is independent of its transporter pathway, thereby providing a potential feedback system driven by the protein’s voltage-induced mechanical activity. A similar non-selective current, GmetL, has been observed along the lateral wall of outer hair cells^19^. Initially, we believed these currents were independent of prestin since the current in outer hair cells had different voltage sensitivity than NLC^17^,^29^. However, our new data require that we reexamine the role of prestin in the non-selective currents observed along the lateral wall of outer hair cells^19^,^29^.

Previous studies have characterized an ionic conductance in the SLC26 transporter family in the presence of SCN^−^. In prestin, we find that this conductance is also demonstrable in the presence of Cl^−^ as the main anion. Our major findings can be summarized as follows. First, there is clear evidence to associate this conductance with prestin: 1) in individual cells the conductance increases with prestin expression and is proportional to NLC. 2) the conductance appears with the same time course as NLC upon release of Golgi block. 3) Mutations in prestin affect the current. An alternative possibility that a prestin associated protein contributes to the current could account for the first two observations. However, the third observation strongly suggests that the current is carried by prestin itself. Moreover, two other SLC26 family members have been demonstrated to have an uncoupled current in the presence of SCN^−^ 14,15.

Our second major finding is that the conductance is carried by a pathway different from the Cl^−^ transport pathway in prestin. Evidence for this is: 1) mutation of residues that are modeled to bind intracellular Cl^−^, the first essential step in the transporter cycle, do not affect the size of the current. 2) salicylate, which in non-mammalian homologs has been shown to block the transporter current while simultaneously blocking NLC, has no effect on the size of the current. 3) single point mutations have effects on the size of the current or on NLC but not both. Since mutation of F137, that we speculate based on homology to UraA to be involved in the transporter cycle, changed the reversal potential in the presence of SCN-, it is possible that the alternative leakage pathway shares some commonality with the transporter pathway. In this context, owing to data that prestin and many similar transporter proteins form dimers and since the charge mutations that affected current are in proximity to its dimer interface, it is possible that the leakage pathway may in fact lie within the dimer interface^45^,^46^,^47^,^48^,^49^,^50^.

The unusual current-voltage relationship can be explained by a channel having a high central energy barrier to ion transport. Consistent with this possibility we note small unitary currents in the fA range. We conclude that prestin forms at least part of an independent pathway for ions to cross the membrane. Mutations suggest that the pathway is located between the core and gate domains of the 7 + 7 inverted repeat structure. In this way the pathway is reminiscent of the omega current pathway in voltage gated channels. There a nonselective cation current is carried by voltage-sensor domains, in a structure entirely separate from the ion-selective pore of these channels.

Our whole cell and patch analyses provide disparate estimates of unitary currents. One explanation could be that only a fraction of prestin molecules show a leakage conductance, as our whole-cell estimates are based on normalization with the total population of prestin molecules derived from NLC. By contrast, the noise analysis provides estimates solely from active channels within a patch (without normalization to NLC as above). We also note that the relative conductances for Cl^−^ and SCN^−^ differed between whole cell recordings and excised patches. These discrepancies between recording approaches could very well be explained by the effects of an increase in membrane tension in excised patches, given the conductance’s tension dependence (Fig. 4E and F).

The non-selective nature of the prestin associated currents and its small size is somewhat at odds given our current understanding of the selectivity filters in ion channels. Perhaps the mechanical model gleaned from structural data of the unrelated EAAT transporter that has a similar leakage conductance (albeit limited to anions) has bearing^51^. In this transporter, the anion leakage pathway is not evident in the crystal structures of the many permutations of the transporter cycle including inside open, outside open and several intermediate states. Molecular dynamics simulations, however, show a conductance pathway that opens several 100 s of nanoseconds after application of voltage. Experimental observations confirm such a pathway. The absence of an anion leakage pathway in the many conformations of the transporter cycle of EAAT crystal structures suggests that the pathway is a transient opening. In prestin, as well, the even smaller size of unitary currents may represent much briefer openings in the leakage pathway. In this respect, the block of the non-selective current in prestin by charge neutralization of both positive and negatively charged residues may result from reduced hydrophilicity, preventing water movement that is modeled to precede ionic movements in EAAT.

Our data suggest that currents carried by prestin’s leakage conductance could affect Cl^−^ in OHCs. A similar conductance in the lateral membrane of OHCs has been demonstrated, and Cl^−^ ions have been shown to affect the voltage sensitivity and affect the speed of transitions from expanded to contracted states of prestin. Although the current carried by individual molecules of prestin is small, the presence of large numbers of prestin in the lateral membrane of OHCs (estimated to be upwards of 10^6^ per OHC) likely makes this current important. Consistent with this possibility, expression of prestin at high levels in HEK cells has a major effect on its resting potential. A similar role has been established in the leakage conductance of EAAT1/2 in glial cells in the cerebellum, that show developmental expression, reducing intracellular chloride^52^.

## Methods

### Cell culture

Chinese hamster ovary (CHO) cells were cultured in Hams-F12 medium (high glucose), containing 50 U/ml each of penicillin and streptomycin, 10% fetal bovine serum, 2 mM L-glutamine at 37 °C in a CO_2_ incubator (5%).

HEK293 cells were cultured in Dulbecco’s modified Eagle’s medium (DMEM, high glucose) containing 50 U/ml each of penicillin and streptomycin, 10% fetal bovine serum at 37 °C in a CO_2_ incubator (5%).

The tetracycline-inducible, highly-expressing monoclonal prestin HEK 293 cell lines were reported previously^28^. Cells were cultured in modified DMEM mentioned above. In addition, 4 μg/ml of blasticidin and 130 μg/ml of zeocin were supplemented in the growth media to maintain prestin expression. Tetracycline (1 μg/ml) was added to the cell growth medium to induce prestin expression. Cells were recorded after 24 to 72 h after tetracycline-induction.

### Mutagenesis

Single amino acid substitutions were generated using QuickChange II site-directed mutagenesis kit (Stratagene, La Jolla, CA) with a gerbil prestin (genbank AF230376^3^)-YFP in pEYFPN1 vector (Clontech, Mountain View, CA) as a template. All mutations were confirmed by DNA sequencing.

### Transient transfection in CHO cells

Transfection of constructs into CHO cells (CHO-K1.ATCC^®^ CCL-61™) was done using Fugene 6 (Promega, Madison, WI) according to the manufacturer’s instructions, in 24-well plates. Cells were recorded 24–72 h after transfection.

### Electrophysiological Recording

Prestin stably expressed in a tetracycline inducible HEK line or transiently transfected into CHO cells were recorded using a whole-cell configuration at room temperature using an Axon 200B amplifier (Molecular Devices, Sunnyvale, CA), as described previously^28^. Cells were recorded 24–72 h after tetracycline induction or transfection to allow for stable measurement of current and NLC. A series of bath solutions with different anions/cations were used. The standard base solution components were (in mM): TEACl 20, CsCl 20, CoCl_2_ 2, MgCl_2_ 2, Hepes 5, pH 7.2. In addition, for anion substitutions the following were added (separately) to the base solution (in mM): NaCl 100, NaSCN 100, NaOCN 100, NaIO_3_ 100, Na Methanesulfonate 100, Na_2_Malate 60, or Na_2_Oxalate 60. Similarly, for cation substitutions the following were added separately to the base solution (in mM): KCl 100, NMDG-Cl 100, or Tris-Cl 100, separately. The pipette solution contains (in mM): NaCl 100/NaSCN 100, CsCl 20, EGTA 5, MgCl_2_ 2, Hepes 10, pH 7.2. Osmolarity was adjusted to 300 ± 2 mOsm with dextrose. After whole cell configuration was achieved in extracellular NaCl and a ramp protocol recorded to confirm baseline NLC and currents. Extracellular anion and cation substitutions were then made by local extracellular perfusion of each individual cell followed by recording a ramp protocol.

Command delivery and data collections were carried out with a Windows-based whole-cell voltage clamp program, jClamp (Scisoft, Ridgefield, CT), using a Digidata 1322A (Axon Instruments).

A continuous high-resolution 2-sine voltage command was used, cell capacitance and current being extracted synchronously. Capacitance data were fitted to the first derivative of a two-state Boltzmann function:


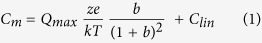


where


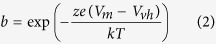


*Q*_max_ is the maximum nonlinear charge transfer, *V*_h_ the voltage at peak capacitance or half-maximal nonlinear charge transfer, *V*_m_ the membrane potential, *C*_lin_ linear capacitance, *z* the unitary charge (a metric of voltage sensitivity), *e* the electron charge, *k* the Boltzmann constant, and *T* the absolute temperature. *Q*_sp_ the specific charge density, is the total charge moved (*Q*_max_) normalized to linear capacitance. Capacitive currents (Icap) generated by our ramp protocol were removed to reveal ionic currents. In order to estimate Icap (from both linear and NLC), we modelled the patch clamp-cell in Matlab and extracted membrane capacitive currents arising from averaged values of linear capacitance and NLC. In all figures, individual I-V plots were corrected based on averages from the inclusive cells. Reversal potentials, thus, arise from ionic current contributions only. Separately, in specific experiments currents were also determined after voltage steps (50 ms duration) from −150 mV to 150 mV, with 20 mV step increments. Where cations and anions were substituted, local perfusion of the cells were estimated to give rise to small junctional potentials (JPCalc function in pClampEx). These varied from +1.1 mV (SCN-) to −5.4 mV (Methanesulfonate) with anion substitutions. Cation substitutions also were estimated to give rise to small junctional potentials varying from −0.8 mV (KCl) to +6.8 mV (NMDG+). Since these numbers were small no corrections were made to the IV plots. For fluid jet experiments we used a QMM perfusion system (ALA Scientific,Instruments, Westbury, NY). The manifold’s output tip was 200 μm placed 1 mm from the cell, and the flow rate increased by an applied pressure of approximately 20 kPa. Significance of the size of currents at different voltage steps from experimental perturbations were determined using a mixed model analysis of correlated measures (MMACM). Statistical analysis was done with SAS software (SAS Institute Inc, NC).

### Inside-out patch recording (noise analysis)

Thick wall glass pipettes were pulled using same puller as described above, and coated with sylgard. The initial resistances of the pipettes are 13–15 mOhm. Pipette solution contains (in mM): NaCl 100, CsCl 20, EGTA 5, MgCl_2_ 2, Hepes 10, pH 7.2. Bath solution contains (in mM):): NaCl/NaSCN 100, TEACl 20, CsCl 20, CoCl_2_ 2, MgCl_2_ 2, Hepes 5, pH 7.2. Osmolarity was adjusted to 300 ± 2 mOsm with dextrose. Inside-out patches were obtained from control HEK cells or the tetracycline-induced prestin stable line. Data acquisition was done with the same interface and amplifier described above. Continuous 10 second commands at desired holding potentials were delivered during recording.

### Noise analysis

PSD analyses of excised patch currents from prestin transfected cells, filtered with a 10 kHz, 4 pole Bessel filter and Hamming windowed, were derived from 10 second recordings held at −50, 0, and +50 mV potentials. In some cases, voltages up to +/−120 mV were analyzed. Some patch records were excluded because of seal loss during recording. Number of patches from separate cells: 11 for symmetrical chloride condition; and 11 for chloride/SCN^−^ conditions 5 for symmetrical chloride of HEK cells and 5 for chloride/SCN^−^ conditions in HEK cells. Spectra were taken for each individual patch recording (1,061,306 pts at 10 μs sampling, with a frequency resolution of 0.095 Hz), averaged and plotted out to 3 kHz. Data are presented following subtraction of baseline spectra at 0 mV. Variance was determined by integration of spectra between 0.1 Hz and 1 kHz after subtraction of calculated shot noise (2ie_0_). Analyses were made with a custom written MATLAB application.


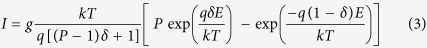


where *g* is the slope conductance at *E* = 0, *q* is the apparent charge, is the electrical distance of the barrier, and *P* is the relative permeability of ions carrying outward current compared to those carrying inward current; if the current were entirely anion selective *P* would be the permeability ratio *P*_SCN_/*P*_Cl_. For Cl^−^ as the external anion the values were P = 1, δ = 0.467, *q* (e_0_) = 0.75 and *g* (pS/pF) = 46.3. For SCN^−^ as the anion the values were P = 6.18, δ = 0.339, *q* (e_0_) = 0.75 and *g* (pS/pF) = 92.8. The values that differ for the two anion species, and the value of *q* smaller than the elementary charge, are consistent with a distribution of barrier locations, for example distinct barrier locations for anions and cations.

## Additional Information

**How to cite this article**: Bai, J.-P. *et al*. Current carried by the Slc26 family member prestin does not flow through the transporter pathway. *Sci. Rep.* **7**, 46619; doi: 10.1038/srep46619 (2017).

**Publisher's note:** Springer Nature remains neutral with regard to jurisdictional claims in published maps and institutional affiliations.

## Supplementary Material

Supplementary Figures

## Figures and Tables

**Table 1:** Effects of extracellular substitution with the pseudohalides OCN^−^ and SCN^−^ on parameters of NLC in tetracycline inducible HEK cell line.

Prestin stable line	*Q*_sp_ (fC/pF)	*V*_h_ (mV)	*z*	n
extracellular Cl^−^	20.32+/−2.21	−96.26+/−3.37	0.74+/−0.02	19
extracellular SCN^−^	15.32+/−1.68	−101.34+/−2.49	0.81+/−0.02	19
extracellular OCN^−^	9.08+/−1.74	−141.72+/−3.94	0.76+/−0.02	6

Specific capacitance was reduced by both extracellular OCN^−^ and SCN^−^ with *V*_h_ shifted to more hyperpolarizing voltages. The effects were more pronounced with OCN^−^. Additionally, SCN^−^ also increased estimates of single charge movement (*z*).

**Table 2:** Noise analysis of inside out patches demonstrate increased variance with SCN^−^ during depolarization.

	Variance, σ^2^ (A^2^)	Mean patch current, <I> (pA)	Estimate of single channel current σ^2^/<I> (fA)	n
−50 mV Cl^−^/Cl^−^	1.66e-26	−11.9+/−5.5	−1.40	11
+50 mV Cl^−^/Cl^−^	2.40e-26	12.6+/−5.6	1.91	11
−50 mV Cl^−^/SCN^−^	1.58e-26	−16.3+/−5.4	−0.97	11
+50 mV Cl^−^/SCN^−^	7.19e-26	14.8+/−5.3	4.87	11

Inside out patches from induced HEK cells expressing prestin were recorded for 10 seconds in each condition. The table lists variance of currents, σ^2^ (A^2^) with symmetrical Cl^−^ and after perfusing with SCN^−^ (with Cl^−^ inside the electrode) at −50 mV and +50 mV. Also shown are mean currents, <I> (re: 0 mV), and estimates (σ^2^/<I>) of single channel currents in each of the conditions.

**Table 3:** Effects of neutralizing charge substitutions along a potential accessory pathway on gating charge parameters on transiently transfected CHO cells.

	*Q*_sp_ (fC/pF)	*V*_h_ (mV)	*z*	n
Prestin (wt)	8.79+/−2.28	−127.69+/−6.31	0.70+/−0.02	8
S398A	4.99+/−0.90	−107.73+/−2.21	0.71+/−0.008	9
**F137A**	—	—	—	5
K227Q	13.08+/−5.32	−146.12+/−6.27	0.67+/−0.03	7
K359Q	5.76+/−0.47	−98.01+/−3.71	0.57+/−0.02	7
R463Q	7.61+/−1.50	−114.32+/−5.62	0.70+/−0.03	9
D485N	6.3+/−1.4	−63.06+/−7.6	1.00+/−0.06	21

Almost all these mutations decreased *Q*_sp_ (except K227Q) and affected the direction of *V*_h_ variably. Effects on unitary *z* were variable and consistent with previous data^20^.
