# Supplementary material for: Current carried by the Slc26 family member prestin does not flow through the transporter pathway
Source: Sci Rep. 2017 Apr 19;7:46619. doi: 10.1038/srep46619 (PMC5395958; doi:10.1038/srep46619)

Current carried by the Slc26 family member prestin does not flow through the transporter pathway

Jun-Ping Bai^*^, Iman Moeini-Naghani^*^, Sheng Zhong^+^, Fang-Yong Li^, Shumin Bian^*^, Fred J. Sigworth^++^, Joseph Santos-Sacchi^+, ++,#^, and Dhasakumar Navaratnam^*,+, #^

Depts. of Neurology*, Surgery^+^, Neuroscience^#^ , Cell and Molecular Physiology^++^, and Yale Center for Analytical Sciences ^, Yale School of Medicine, 333 Cedar Street, New Haven, CT 06510.

Corresponding author- Dhasakumar Navaratnam Dhasakumar.Navaratnam@Yale.Edu

Supplementary Figure 1.

Extracellular salicylate (20mM) has no effect on currents (**A**) of prestin expressing cells in the presence of extracellular Cl^-^ , while reducing NLC (**B**). Shown are average currents (+/- SEM) and corresponding NLC from 8 prestin expressing cells (A and B) and four control cells (A).

Supplementary Figure 2.

The effects of fluid-jet pressure on the size of the current in control HEK cells while varying the conducting anion. With Cl^-^ in the bath solution there is a smaller increase in the size of the current compared to prestin expressing cells with exposure of the cell to a similar fluid jet (A). In the presence of extracellular SCN-, the size of the current shows a smaller decrease compared to prestin expressing cells exposed to a similar fluid jet (B). Shown are average tracings from 10 cells (+/-SEM).

Supplementary Figure 3. Shown is the alignment of amino acid sequences of prestin (genbank AF230376) and UraA (Protein Data Bank (PDB) code 3QE7) using HHPred limited to prestins transmembrane segment. Conserved residues are marked with a / sign and conservative substitutions indicated with a + sign. Also shown is the predicted secondary structure of prestin that is compared to the known secondary structure of UraA.The order of alignments is as follows: prestin predicted secondary structure (SLC26 SSP), prestin amino acid sequence, conserved sequence, UraA amino acid sequence (3QE7 AA), UraA secondary structure (3QE7 SS)

Supplementary Figure 4. Shown are four representative confocal images of CHO cells transiently transfected with F137A-prestin YFP 48 hours after transfection. Cells were fixed in 4% PFA for 30 mins and imaged with a Zeiss spinning disk confocal microscope. YFP was excited at 488nm and YFP fluorescence detected after filtering emitted light with a 500-550nm bandpass filter. As is evident F137A Prestin YFP is targeted to the membrane of the cell. The scale bar is 10 microns.

Supplementary Figure 1


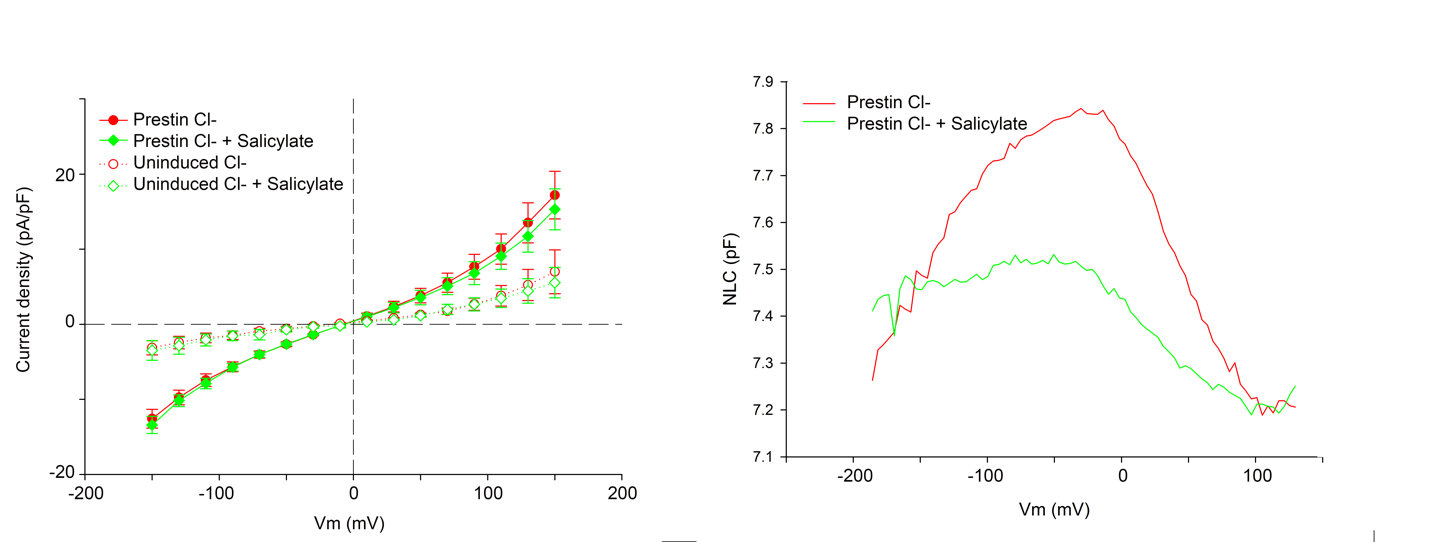


Supplementary Figure 2


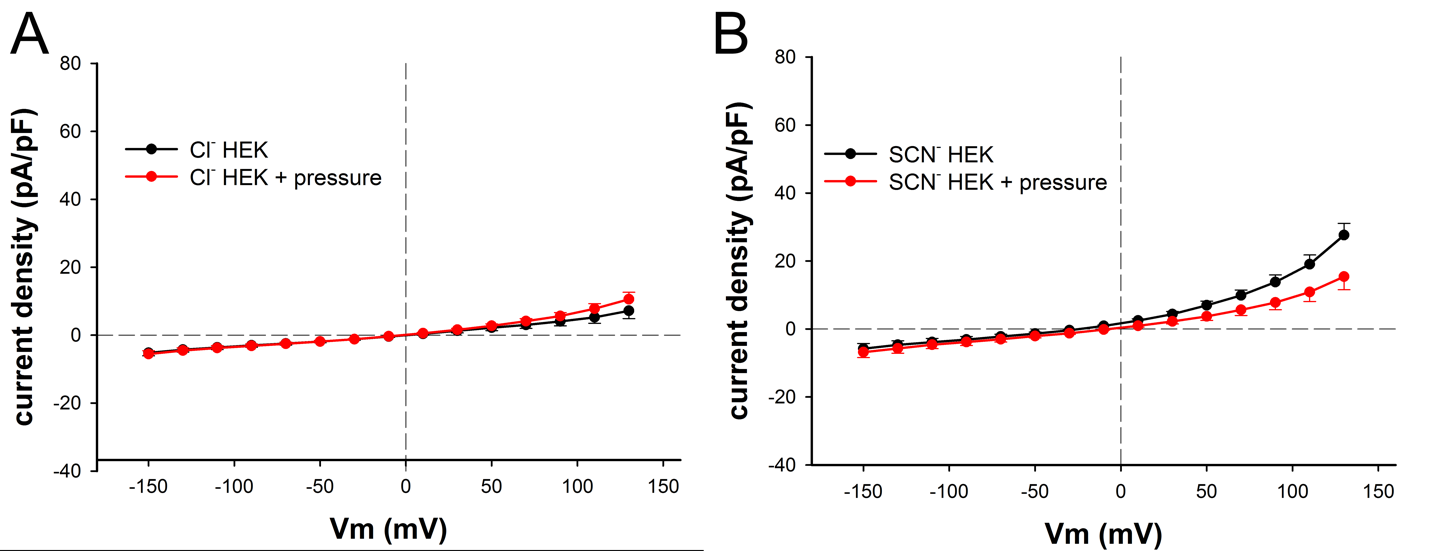


Supplementary Figure 3

Supplementary Figure 4


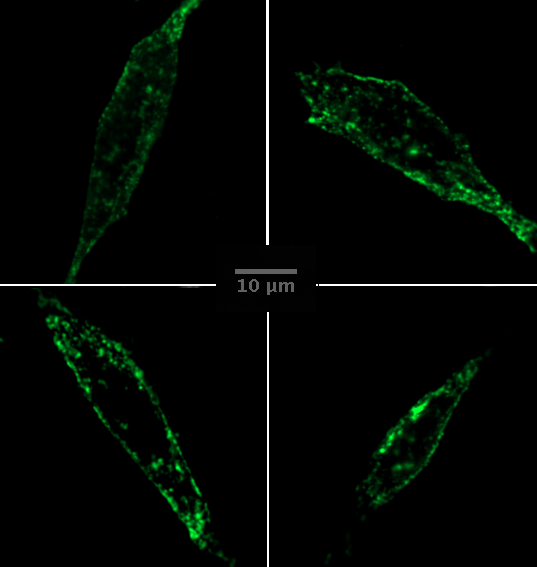

Supplement: Supplementary Figures [file srep46619-s1.doc]
